# Mitral Valve Prolapse in a Patient With Polycystic Kidney Disease

**DOI:** 10.7759/cureus.72931

**Published:** 2024-11-03

**Authors:** Leticia Santos, Filipa Monteiro, Ana C Gomes, Paula S Fazendas, Helder H Pereira

**Affiliations:** 1 Internal Medicine, Hospital Garcia de Orta, Almada, PRT; 2 Cardiology, Hospital Garcia de Orta, Almada, PRT

**Keywords:** autosomal dominant polycystic kidney disease (adpkd), heart faillure, mitral valve prolapse, renal cysts, surgical valve repair

## Abstract

Autosomal dominant polycystic kidney disease (ADPKD) is a multisystemic heterogeneous disease characterized by the presence of cysts in several organs leading to progressive dysfunction. The cardiovascular manifestations of ADPKD include hypertension, left ventricular hypertrophy, and valvular heart disease, predominantly mitral valve abnormalities. We present the case of a 30-year-old male with a past medical history of ADPKD who was admitted to the emergency department due to sudden chest pain and signs of congestive heart failure for weeks. Echocardiography in the emergency department showed lateral wall hypokinesis and severe mitral regurgitation. Coronary angiography revealed a small collateral branch occlusion unsuitable for revascularization.

On the first day of hospitalization, the patient developed an acute ischemia of the left lower limb, for which he underwent revascularization surgery. After the thrombectomy, the patient presented with fever, for which prophylactic antibiotics were started while awaiting investigation into surgical and cystic complications, such as infection, which were ruled out. Once stabilized, a second transthoracic echocardiogram confirmed the severe mitral regurgitation and prolapse due to posterior mitral valve flail, suggesting long-term primary mitral valve disease as the underlying mechanism for regurgitation. The patient underwent surgical mitral valve repair, which was complicated by suture dehiscence.

The severe mitral valve regurgitation was attributed to ADPKD, given the patient's family history, age, and typical cardiovascular findings and multiple renal and hepatic cysts observed. Further investigation into primary mitral valve disorders, such as soft connective tissue diseases like Marfan syndrome, was not deemed necessary.

## Introduction

Autosomal dominant polycystic kidney disease (ADPKD) is estimated to affect over 10 million people globally [1] and its calculated prevalence in the European Union is 3.96/10,000 [2]. It is the most prevalent inherited progressive kidney disease and the fourth most common cause of end-stage kidney disease [3]. ADPKD is a genetically and phenotypically heterogeneous disease. Mutations in the PKD1 gene account for 85% of cases (located on chromosome 16), while PKD2 mutations account for the remaining 15% of cases (located on chromosome 4), encoding the polycystin-1 or -2 proteins, involved in various organ development such as smooth muscle cells, myofibroblasts of the tunica media, and the endothelial layer of vessels [3-6]. PKD2 is usually associated with a more benign course, with the formation of fewer cysts, lower kidney volume, and later onset of renal failure with impaired concentrating capacity [3-4,7].

ADPKD manifestations are typically categorized as cystic and non-cystic, or renal and extrarenal. The hallmark of ADPKD is the development of multiple fluid-filled cysts within the kidneys, leading to increased kidney volume and impaired renal function [1,3-5]. ADPKD is the most likely diagnosis in patients presenting with bilaterally enlarged kidneys and more than 10 cysts in each kidney [5-6]. Focusing on personal and family history of complications and symptoms (namely the most frequent, such as abdominal fullness and mass, lumbar pain, haematuria, and urinary tract infections) is essential for an accurate diagnosis [3,6]. In up to 90% of recently diagnosed ADPKD cases, a family history of ADPKD or complications such as early sudden death or intracranial aneurysm rupture will be present [6,8]. Therefore, ADPKD diagnosis relies on family history, the patient’s age, and typical imaging findings, such as the presence of more than 10 cysts in those aged 16-40 years [3,6]. In this report, we discuss the case of a male patient with a past medical history of ADPKD.

## Case presentation

The patient was a 30-year-old male, a smoker with a history of renal lithiasis and ADPKD, who had been diagnosed at the age of 21 years and inherited maternally. He had not undergone any previous routine cardiovascular evaluation since his diagnosis. The patient was admitted to the emergency department with complaints of intermittent chest pain for the past two hours. He also reported progressive fatigue, shortness of breath, and peripheral edema over the last two months. On examination, he appeared tachypnoeic, was normotensive (blood pressure of 110/80 mmHg), and had a tachycardic heart rate (of 110 bpm). Auscultation revealed a III/VI systolic murmur most audible at the lower left sternal border. Additional findings included bilateral lung crackles, bilateral lower limb pitting edema, and mild ascites, compatible with heart failure of New York Heart Association (NYHA) class III, hemodynamic profile B.

**Figure 1:**
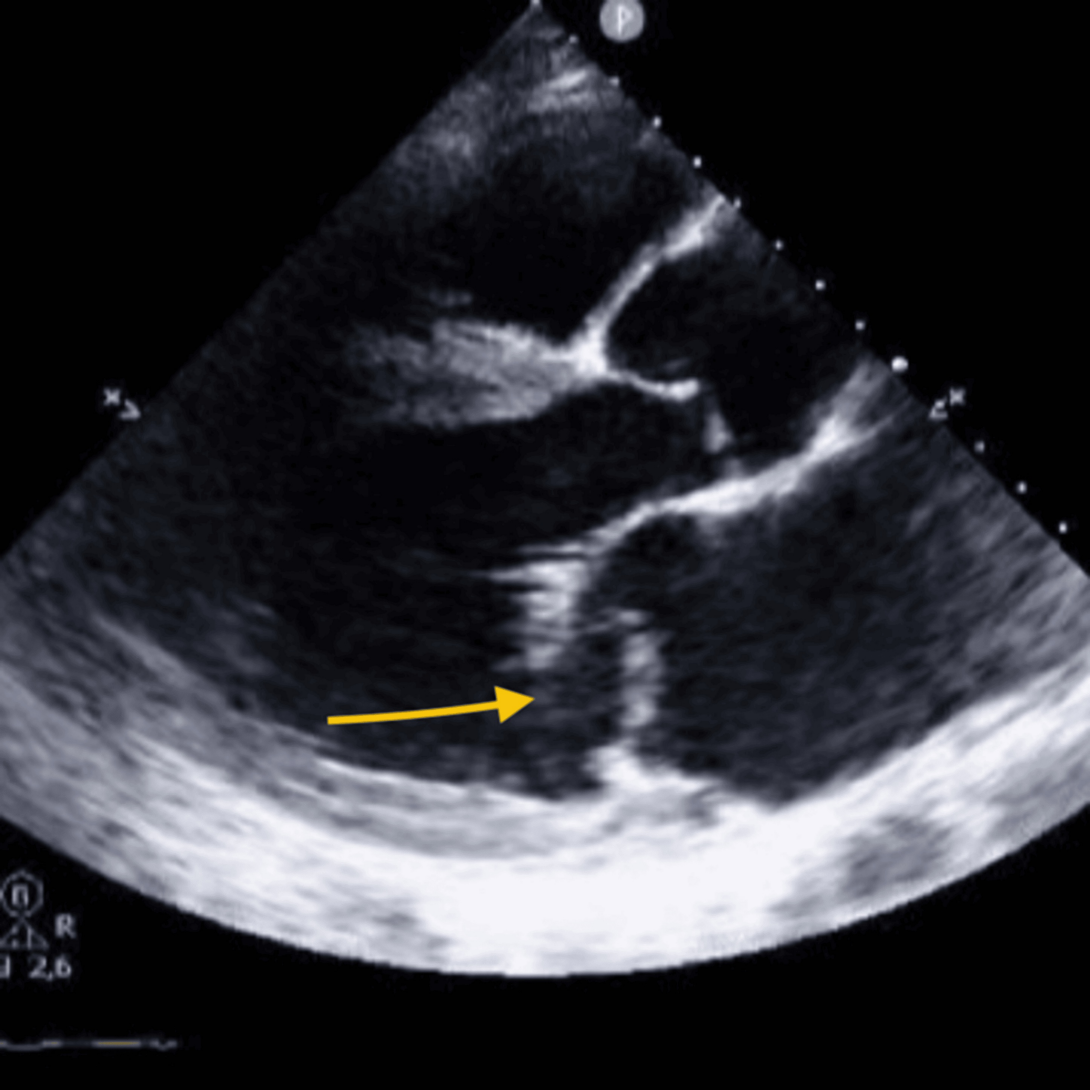
Transthoracic echocardiogram parasternal long-axis view revealing a major mitral regurgitation

**Figure 2:**
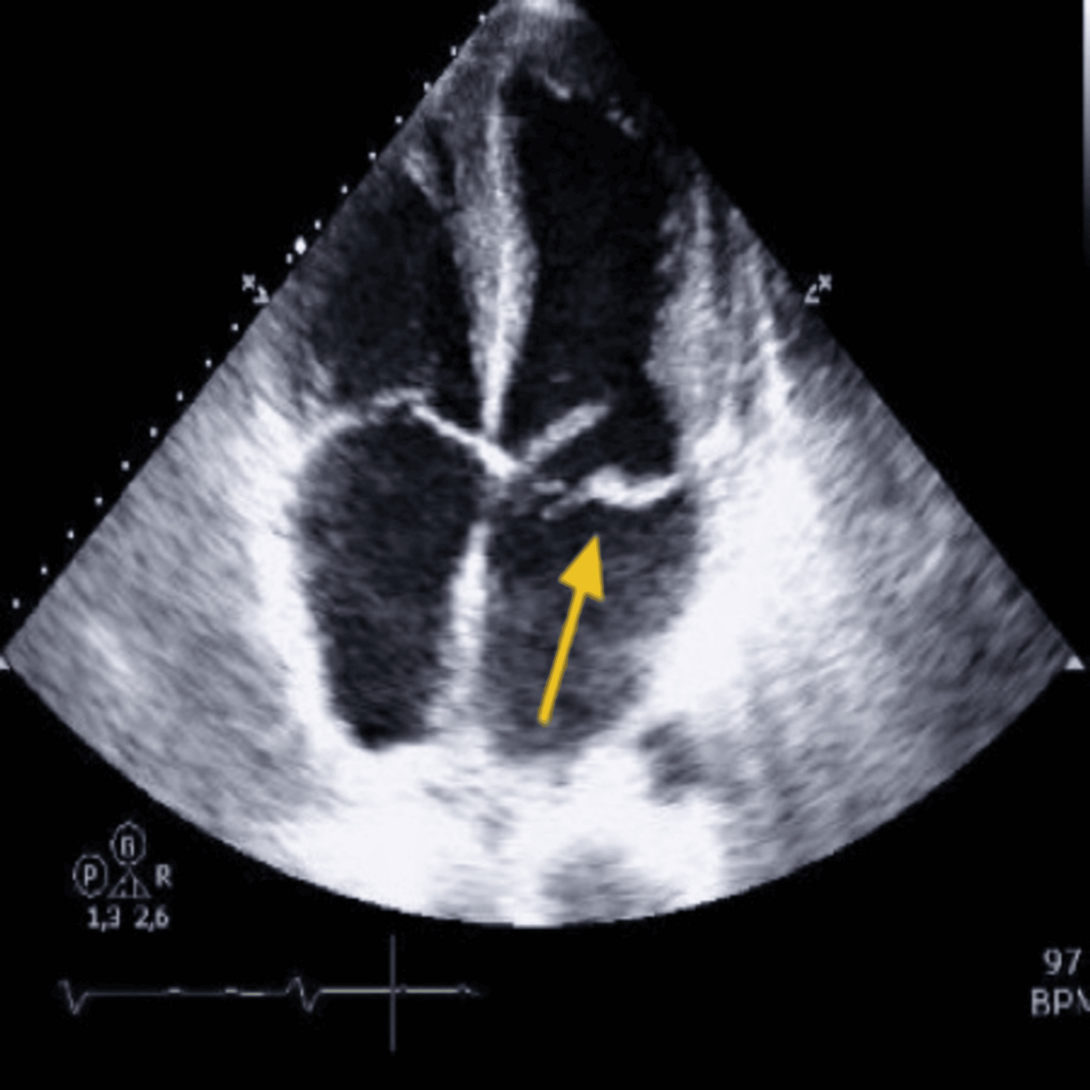
Transthoracic echocardiogram apical four-chamber view revealing a major mitral regurgitation, mild tricuspid regurgitation, and a preserved global systolic function

**Figure 3:**
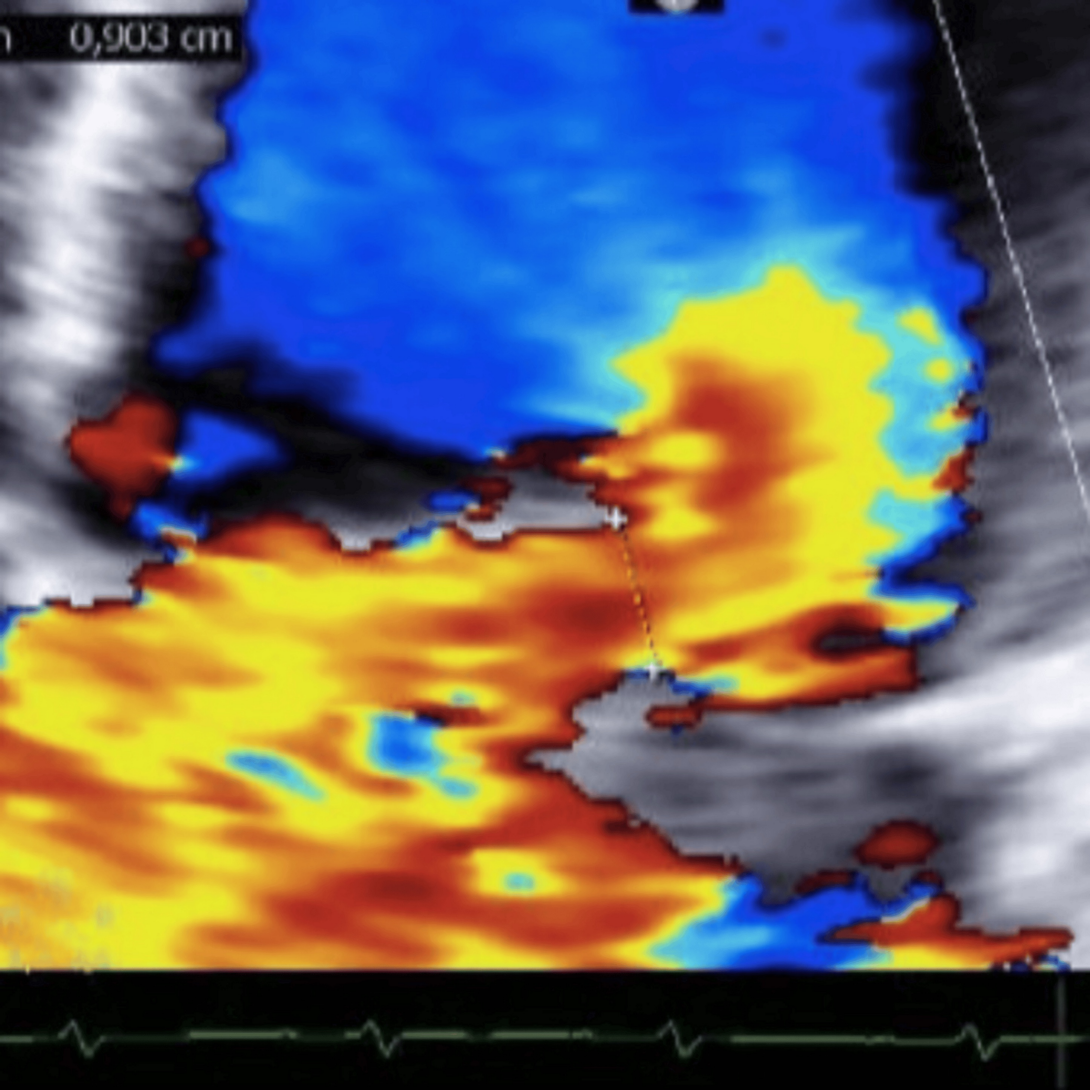
Transthoracic echocardiogram apical four-chamber view revealing a major mitral regurgitation with an antero-medially directed jet and of unclear mechanism

The electrocardiogram showed sinus tachycardia with 0.1 mV ST-segment elevation in leads aVL, DI, and V6, and T-wave inversion in lead III. Arterial blood gas analysis revealed compensated respiratory alkalosis with hypoxemia (pH 7.43, PaCO_2_ 25 mmHg, PaO_2_ 63 mmHg) and lactate within the normal range, suggesting that tissue oxygenation remained likely adequate despite the hypoxemia. Blood tests were largely unremarkable, except for an elevated high-sensitivity T-troponin (maximum value of 1760 ng/L, against a normal range of <14 ng/L) and a creatinine of 1.4 mg/dL. An urgent focus transthoracic echocardiogram (Figures 1-3) showed hypokinesis of the mid and apical lateral wall, severe mitral regurgitation with an antero-medially directed jet, and mild tricuspid regurgitation, while global systolic function was preserved. The mechanism of mitral regurgitation was unclear: either chordal rupture in primary mitral valve disease or partial papillary muscle rupture due to an acute coronary syndrome (lateral STEMI). Emergent coronary angiography was performed, revealing a lesion in a small collateral of the marginal branch, unsuitable for revascularization.

A second transthoracic echocardiogram (Video 1) confirmed severe mitral regurgitation and prolapse due to posterior mitral valve flail (with posterior leaflet exhibiting unrestricted outward motion, concave toward the left atrium like a parachute). Additional findings included pulmonary hypertension (pulmonary arterial systolic pressure of 58 mmHg), left and right atrial enlargement, and a dilated left ventricle with preserved systolic function. These findings suggested a long-term primary mitral valve disease as the underlying mechanism behind regurgitation.

**Video 1:** Transthoracic echocardiogram apical four-chamber view revealing a major mitral regurgitation with an antero-medially directed jet and of unclear mechanism

**Figure 4:**
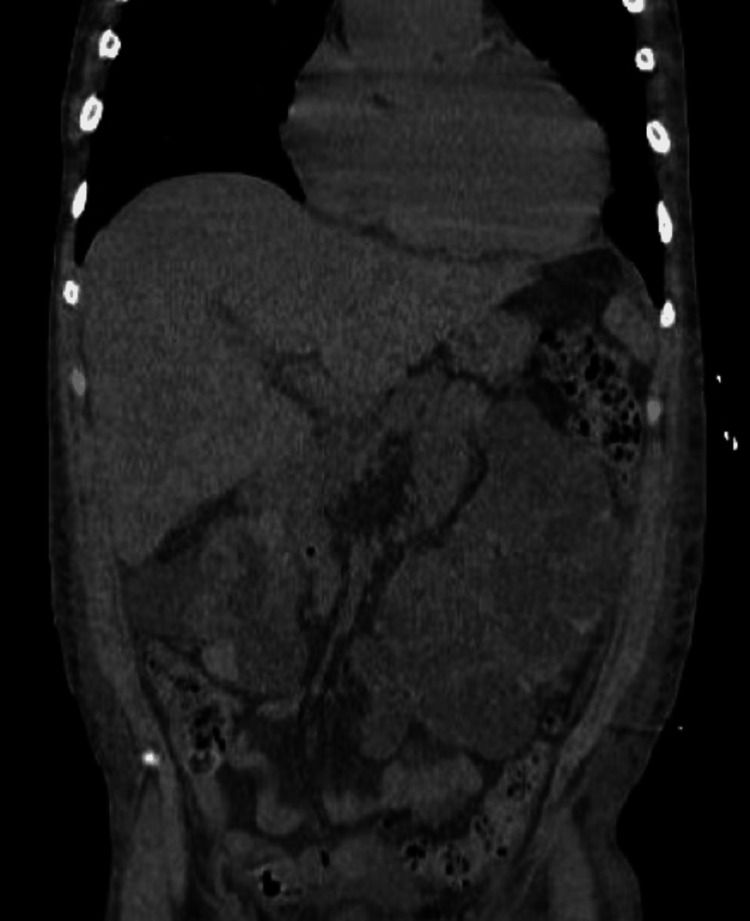
Contrast-enhanced CT scan of the abdomen and pelvis performed to rule out post-surgical and cystic complications revealing multiple renal and hepatic cysts with no evidence of infection, bleeding, or obstruction CT: computed tomography

During the first 24 hours of hospitalization, the patient experienced fever and sudden pain in the left lower limb, which became pale and cold. An echo Doppler study showed acute iliofemoral arterial thrombosis and an emergent thrombectomy was successfully performed. In the next few days, the patient presented with persistent fever. Given the occurrence of a second arterial thrombotic/embolic event, prophylactic antibiotics were initiated after the collection of multiple blood cultures (to mitigate potential false negatives due to low bacterial load or fastidious organisms). A contrast-enhanced CT scan of the abdomen and pelvis was also performed to rule out post-surgical and cystic complications, revealing multiple renal and hepatic cysts (Figure 4) with no evidence of infection, bleeding, or obstruction.

The transesophageal echocardiogram (Video 2) showed severe mitral regurgitation, no clear evidence of endocarditis, no papillary muscle abnormalities, and a patent foramen ovale with a left-right shunt. Despite several sets of sterile blood cultures, a PET scan was performed, which was unremarkable for ongoing endocarditis. Finally, the culture of the surgical material confirmed a sterile thrombus, ruling out acute non-thrombotic embolization.

**Video 2:** Transesophageal echocardiogram revealing a severe mitral regurgitation and no evidence of endocarditis or papillary muscle abnormalities

The patient was successfully managed with high doses of diuretics and vasodilators, with partial improvement of congestive symptoms, while awaiting surgery in the cardiac care unit. He underwent surgical mitral valve repair with a triangular posterior leaflet resection and a 34 mm Carpentier-Edwards Physio IITM ring (Edwards Lifesciences Corp., Irvine, CA). Tricuspid annuloplasty with a 34 mm Edwards MC3TM ring (Edwards Lifesciences Corp., Irvine, CA) and closure of patent foramen ovale were also performed. The immediate operative result was satisfactory but was complicated by suture dehiscence a few days later, requiring reoperation, which was successful. 

In ADPKD patients, surgery may be complicated due to tissue fragility, impaired kidney function, and fluid overload, necessitating careful surgical planning and perioperative management. These patients have a higher risk of complications, such as suture dehiscence, related to connective tissue abnormalities impacting blood vessels, heart valves, and even the pericardium. The patient's in-hospital recovery after surgery involved close monitoring of arrhythmias, heart failure, and fluid overload symptoms, as well as kidney function and echocardiogram evaluations. Diuretics and fluid restriction were not required at discharge, since the patient was asymptomatic. At discharge, the patient was advised to monitor his blood pressure, moderate sodium intake, and avoid intense activities for the first few months post-surgery. He was instructed to seek emergency care for any concerning symptoms, such as signs of heart failure, arrhythmias, neurological and kidney dysfunction symptoms, and fever.

The follow-up care for the patient included coordination between cardiologists and nephrologists consultations, as well as echocardiographic assessment and laboratory analysis, including creatinine and electrolytes, which were scheduled for the first, three, and sixth months in the first year following surgery. The postoperative echocardiogram showed no residual mitral valve regurgitation, no pulmonary hypertension, and normal biventricular systolic function, and the patient remained asymptomatic during the first 12 months of follow-up consultations in our valvular heart disease clinic.

## Discussion

In ADPKD, cysts are usually simple and solitary, and can also be seen in various organs, most often the liver, in about 80% of patients by 30 years of age (but also pancreas, spleen, thyroid, ovaries, seminal vesicles, meninges, and arachnoid membrane), as well as their complications (such as infection, rupture and bleeding, extrinsic compression and thrombosis of the vena cava, male infertility, and predisposition to kidney stone formation in up to 35% of cases [1,3-5,8-9]. Non-cystic manifestations encompass cardiac, vascular, and connective tissues, including cardiovascular, diverticulitis, abdominal hernias, and bronchiectasis [3-6, 8-9]. The extrarenal and renal manifestations (including kidney disease severity) are quite variable and strongly influenced by phenotypic variability and genetic heterogeneity, with PKD1 patients being documented to suffer from end-stage renal disease 20 years before PKD2 patients [8,10]. Due to phenotypic and genetic variability among disorders that cause PKD, including ADPKD, tuberous sclerosis complex, von Hippel-Lindau disease, and autosomal dominant tubulointerstitial kidney disease, gene panel screening may be essential sometimes [6].

​​​The prevalence of cerebral aneurysms in ADPKD patients is up to fivefold higher than in the general population (and triples when family history is present, due to familial clustering), with rupture being fatal in up to 50% of cases and disabling at young ages (often around 40s) [3,5,9-11]. The most consistent strong risk predictor for intracranial aneurysm rupture is its size, which highlights the need for early pre-symptomatic screening for family members at risk and intervention protocols, particularly when family history is present or before major surgery [3,6,10,12]. A wide spectrum of cardiovascular abnormalities is often seen in patients with ADPKD, and these are now the leading cause of death; these include hypertension, valvular abnormalities, cardiomyopathy, and cerebral aneurysms [10-13]. Their prevalence is very variable, which can be as high as 30% of cases, probably due to the lack of genotyping patients into subgroups (since patients with PKD1 seem to have more severe disease than patients with PKD2) [3]. 

The most common cardiovascular complication of ADPKD is hypertension, which has an average age of onset of 30 years (up to 70% of patients experience it before renal function decline) and is associated with left ventricular hypertrophy, cardiovascular death, and renal function worsening [3,6,11-12]. Blood pressure control, with angiotensin-converting enzyme inhibitors or angiotensin II receptor blockers, is critical to prevent cardiac dysfunction and complications, as well as mortality [3,6,12]. Cardiovascular abnormalities, such as valvular heart disease, are the leading cause of death in patients with ADPKD [3,12]. Cardiac involvement in ADPKD most commonly manifests a mitral valve prolapse (MVP) in approximately 25% of cases (compared to a 2-3% prevalence in the general population), and mitral regurgitation in up to 31% of cases; but other abnormalities can also be seen, such as regurgitation of other valves, aortic dilata­tion and dissection, coronary aneurysm and dissection, pericardial effusion and cardiomyopathy [3-4,10-12,14]. These findings highlight the importance of cardiac screening to facilitate early detection and management, aiming to improve ADPKD patient outcomes [10,12].

Mechanisms underlying cardiovascular defects are still being studied; they include reduced expression of the polycystin proteins in the endothelial and vascular smooth muscle cells, mechanisms including disruptions in vascular tone regulation, collagen and connective tissue abnormalities, and overactivity of the renin-angiotensin-aldosterone and sympathetic nervous systems [1,6,10-11]. PKD1 and PKD2 patients seem equally likely to develop intracranial aneurysms, while patients with specific mutations of PKD1 are more likely to have vascular complications [10]. MVP is often observed in PKD1 patients due to extracellular matrix abnormalities in the mitral valve, which predispose it to prolapse, particularly in younger individuals, which increases the risk of mitral regurgitation, primarily due to elevated blood pressure, with contributing factors such as left ventricular hypertrophy, dilation of the mitral annulus, and calcification [3,11,14]. MVP, especially associated with mitral regurgitation, can present with potentially severe symptoms of heart failure.

Current treatment includes lifestyle changes (to enhance hydration, control sodium and protein intake, and exercise), symptom management ​(more frequently, pain management), cardiovascular risk factor control (to reduce disease progression and renal function decline), and addressing complications (ultimately involving replacement by dialysis or transplant) [3,6,12,15]. In recent years, tolvaptan - a vasopressin V2 receptor antagonist and the first drug approved specifically for the treatment of ADPKD - has become an important treatment option for decreasing renal decline in patients with rapid disease progression [6,15]. The Mayo Clinic ADPKD classification scheme is one of the screening tools used to predict future progression, using total kidney volume as a prognostic factor [3,15]. Inhibition of molecular cascades promoting epithelial cell proliferation or reducing intracellular cyclic adenosine monophosphate (cAMP) levels have been targeted in studies to address cardiovascular abnormalities, such as mTOR inhibitors or somatostatin analogs [3]. Future research should focus on advanced imaging techniques to enable earlier diagnosis, genetic sequencing for precision medicine, and new therapies targeting cyst formation [6,15].

For an ADPKD patient presenting with fever and acute arterial thrombosis following heart catheterization, the approach must be thorough and proactive given the heightened risk for infectious and thromboembolic complications. These pose significant concerns in ADPKD patients due to valvular disease, the immunocompromised state often seen in ADPKD and kidney disease, and the risk of bacteremia​​ associated with the intervention. Fever in combination with a newly formed arterial thrombus also raises concerns about a possible septic embolus. Endocarditis with multiple embolization events would explain the whole case, which is a potentially life-threatening complication if not promptly treated. In this scenario, waiting for culture results was deemed risky (due to the risk of valvular destruction, abscess formation, or septic emboli), and hence proactive empiric antibiotics were started while awaiting further diagnostic clarity due to the patient’s high-risk profile.

Echocardiography did not any show signs of endocarditis, and the thoracic, abdominal, and pelvic contrast-enhanced CT scan ruled out intervention and cystic complications. Besides several sets of sterile blood cultures, we performed a PET scan, which was negative for endocarditis, and no other source of infection was identified. The hypothesis was abandoned, and antibiotic treatment was suspended. Arterial thrombosis shortly after catheterization raises concern for a systemic or localized prothrombotic state, potentially triggered by infection, endothelial injury from catheterization, or vascular fragility and inflammation associated with ADPKD. In the setting of severe atria dilation (causing blood stasis, endothelial dysfunction, and increased risk of atrial fibrillation), there could have been a minor episode of atrial fibrillation with thrombus formation and peripheral embolization, which could explain both events.

The other contributing factors that we considered included auto-immune disease (such as vasculitis, antiphospholipid syndrome, and systemic lupus erythematosus) or hypercoagulable states (either primary as genetic thrombophilias or secondary associated with malignancy, inflammation, or chronic diseases). A multidisciplinary discussion was conducted to look into these quite remote hypotheses, in comparison with the previous ones, given the lack of any relevant history, evidence, or typical symptoms. Furthermore, the timing of symptoms in the context of recent catheterization raises a higher suspicion for more localized vascular injury and infection, which is a more immediate concern given the patient’s high-risk profile, guiding the clinical decision to prioritize empiric antibiotic therapy and to focus on infectious workup.

Given the patient's family history, age, and overall imaging findings (specifically related to renal and cardiac assessments), a diagnosis of ADPKD was established. Further investigation into primary mitral valve disorders, such as soft connective tissue diseases like Marfan syndrome, was deemed unnecessary. Echocardiographic periodic monitoring to assess valve function, ventricular size, and overall cardiac performance is critical since the re-emergence of regurgitation will impact both cardiac and kidney function. Systematic screening of intracranial aneurysms is not recommended and was not done in our patient since there was no personal or family history of neurologic events; however, it should be considered if the patient undergoes major surgery in the future.

## Conclusions

ADPKD is the most prevalent inherited progressive kidney disease, driven primarily by mutations in the PKD1 and PKD2 genes. The characteristic gradual growth of fluid-filled cysts in the kidneys leads to kidney enlargement and progressive chronic kidney disease, resulting in end-stage renal disease in about half of the patients by the age of 60. Vascular changes, such as endothelial dysfunction and increased carotid intima-media thickness, suggest that atherosclerosis is a very early event in the course of the disease, with cardiovascular complications being the leading cause of death in ADPKD patients. The most common cardiovascular complication of ADPKD is hypertension (average age of onset: 30 years) and cardiovascular abnormalities like valvular heart disease. Cardiac involvement most commonly manifests as mitral valve abnormalities, such as prolapse and incompetence, as in our patient. MVP can potentially cause varying degrees of mitral regurgitation and severe symptoms of heart failure.

Systemic complications like infection and thrombosis can rapidly lead to severe outcomes in ADPKD patients, especially due to vascular fragility and underlying structural abnormalities. This case shows the importance of a multidisciplinary approach and highlights how connective tissue abnormality can predispose ADPKD patients to surgical complications. Being an incurable, complex, and multisystemic condition, increased awareness of cardiovascular risks in ADPKD, which enables early diagnosis, is essential to control cardiovascular risk, promptly address complications, and improve overall prognosis.
